# Automated detection of archaeological mounds using machine-learning classification of multisensor and multitemporal satellite data

**DOI:** 10.1073/pnas.2005583117

**Published:** 2020-07-20

**Authors:** Hector A. Orengo, Francesc C. Conesa, Arnau Garcia-Molsosa, Agustín Lobo, Adam S. Green, Marco Madella, Cameron A. Petrie

**Affiliations:** ^a^Landscape Archaeology Research Group (GIAP), Catalan Institute of Classical Archaeology, 43003 Tarragona, Spain;; ^b^Institute of Earth Sciences Jaume Almera, Spanish National Research Council, 08028 Barcelona, Spain;; ^c^McDonald Institute for Archaeological Research, University of Cambridge, CB2 3ER Cambridge, United Kingdom;; ^d^Culture and Socio-Ecological Dynamics, Department of Humanities, Universitat Pompeu Fabra, 08005 Barcelona, Spain;; ^e^Catalan Institution for Research and Advanced Studies, 08010 Barcelona, Spain;; ^f^School of Geography, Archaeology and Environmental Studies, The University of the Witwatersrand, Johannesburg 2000, South Africa;; ^g^Department of Archaeology, University of Cambridge, CB2 3DZ Cambridge, United Kingdom

**Keywords:** multitemporal and multisensor satellite big data, machine learning, archaeology, Indus Civilization, virtual constellations

## Abstract

This paper presents an innovative multisensor, multitemporal machine-learning approach using remote sensing big data for the detection of archaeological mounds in Cholistan (Pakistan). The Cholistan Desert presents one of the largest concentrations of Indus Civilization sites (from *ca*. 3300 to 1500 BC). Cholistan has figured prominently in theories about changes in water availability, the rise and decline of the Indus Civilization, and the transformation of fertile monsoonal alluvial plains into an extremely arid margin. This paper implements a multisensor, multitemporal machine-learning approach for the remote detection of archaeological mounds. A classifier algorithm that employs a large-scale collection of synthetic-aperture radar and multispectral images has been implemented in Google Earth Engine, resulting in an accurate probability map for mound-like signatures across an area that covers *ca*. 36,000 km^2^. The results show that the area presents many more archaeological mounds than previously recorded, extending south and east into the desert, which has major implications for understanding the archaeological significance of the region. The detection of small (<5 ha) to large mounds (>30 ha) suggests that there were continuous shifts in settlement location. These shifts are likely to reflect responses to a dynamic and changing hydrological network and the influence of the progressive northward advance of the desert in a long-term process that culminated in the abandonment of much of the settled area during the Late Harappan period.

## Significance

This paper illustrates the potential of machine learning-based classification of multisensor, multitemporal satellite data for the remote detection and mapping of archaeological mounded settlements in arid environments. Our research integrates multitemporal synthetic-aperture radar and multispectral bands to produce a highly accurate probability field of mound signatures. The results largely expand the known concentration of Indus settlements in the Cholistan Desert in Pakistan (*ca*. 3300 to 1500 BC), with the detection of hundreds of new sites deeper in the desert than previously suspected including several large-sized (>30 ha) urban centers. These distribution patterns have major implications regarding the influence of climate change and desertification in the collapse of the largest of the Old-World Bronze Age civilizations.

Artificial mounds are a characteristic feature of permanent and semipermanent settlement locations in past cultural landscapes, particularly on sedimentary plains, but also in arid and semiarid regions. These mounds can be readily visible due to their prominence and shape, and the fact that they are composed of accumulated debris such as mud bricks and pottery sherds, which creates specific soil with distinct color and surface texture. These characteristics make them detectable using different methods, and their number and distribution have seen them play an important role in addressing questions about the formation of early urbanism, states, and economic systems.

The use of remote sensing (RS) to detect and map archaeological mounds has been attempted in many parts of the world (1–4). Much research has focused on arid and semiarid areas in the Levant and the Near East, where the geomorphological and sedimentary properties of mounds make them highly visible in digital elevation models and aerial and satellite imagery (5). Mounds can also leave specific multispectral soil signatures in highly anthropized landscapes with leveled or irrigated fields (6). When available, the use of declassified historical photographs such as CORONA imagery has been critical to the detection of mounds (7–9). Georeferenced historical map series have also been used solely or in combination with contemporary declassified data (10–14). In recent years, RS-based archaeological research has gradually incorporated machine-learning techniques and algorithms that facilitate the automated detection of sites and features. Most of those applications have focused on the detection of small-scale features using high-resolution datasets such as lidar (15) or WorldView imagery (16–18). In the Near East, Menze and Ur (19) applied a random forest (RF) classifier over a multitemporal collection of ASTER imagery to identify probable anthrosols. Some other attempts have used multitemporal data to monitor archaeological sites and human impact such as urban sprawl and looting (20–22). The detection of anthropic signatures, such as those that characterize mounded sites, across a very large area, remains seldom attempted, presumably due to the large computational resources, coding expertise, and large amount of satellite data required.

This paper presents a machine-learning approach for the detection of mounded sites across one such very large area. It employs multitemporal data in a way similar to the approach used by Menze and Ur (19), but rather than detecting anthroposols, which include modern towns, settlements, and other areas of human use or occupation, this study outlines a method for restricting the algorithm detection to archaeological mounds. It does this by employing a multisensor and multitemporal approach that combines synthetic-aperture radar (SAR) data and multispectral satellite imagery. The study area is the Cholistan Desert in Pakistan, which is a large arid area that has long been considered a core region of South Asia’s Indus Civilization (*ca*. 3300 to 1500 BC). The results of the analysis are evaluated, compared to previous field survey data, and discussed in relation to the prevailing interpretations of the trajectories of settlement in the region, including the development and decline of the large urban centers of the Indus Civilization.

## Research Background

### The Cholistan Desert and the Indus Civilization.

**Fig. 1.**
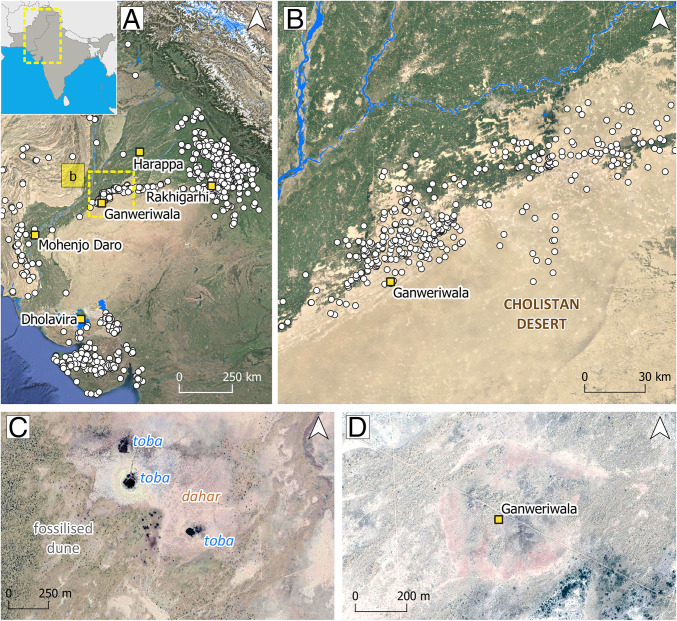
Location map of the Cholistan Desert. (*A*) Distribution of Indus sites with the location of the five major Indus cities during the urban period (*ca*. 2500 to 1900 BC). (*B*) Distribution of Indus sites in the Cholistan Desert, after Mughal (26, 47). (*C*) Inset showing characteristic fossilized sand dunes, mud flats (*dahars*), and water ponds (*tobas*). (*D*) The well-known mound of Ganweriwala, partially covered by a sand dune.

The Cholistan Desert is the western extension of the Great Indian or Thar Desert and stretches from the southern edge of the alluvial plains of Punjab to the north of Sindh province in Pakistan (Fig. 1 *A* and *B*). The area is usually described as a marginal arid region that is highly sensitive to the annual variation of the Indian summer monsoon (23), the intensity of which has significantly affected its population and ecological diversity throughout the Holocene (24). Today its landscape is characterized by fossilized sand dunes with shrub vegetation, small patches of trees around artificial water tanks, called *tobas,* and saline mud flats called *dahars* (ref. 25 and Fig. 1*C*). The region has played a significant historical role in transcontinental mobility between central and South Asia, as attested by the caravan routes that crossed the area and the numerous forts that protected them (26). The area has also been home to nomadic pastoralists who have moved with their livestock near *tobas* (23, 27). In recent decades, however, major investment in irrigation schemes in the western plains has changed the traditional subsistence strategies and movement patterns of local populations (28–30). Cholistan has figured prominently in discussions about the Indus Civilization, and Possehl (31, 32) argued that it was the most important area of settlement concentration during the Mature Harappan period (also Harappa phase; *ca.* 2500 to 1900 BC), which is when South Asia’s first cities flourished. There has been considerable discussion about the region’s hydrological network and its relation to the former Ghaggar-Hakra River, and the date at which it ceased to flow perennially is much debated (33–38). Despite its perceived archaeological importance, at present only survey data are available for the region.

The first archaeological explorations in Cholistan can be traced back to the early European officials operating in the area in the 1830s and 1840s, when sporadic mounded sites were reported in traveler’s notes (39, 40). Oldham (41) was among the first explorers to report scattered mounded sites in the desert in the late 1890s, and these observations were reiterated in the 1940s and 1950s by Stein (42) and Field (43). The most extensive work in the region was led by Mughal, who executed an extensive survey between 1974 and 1977 (26). Additional areas of Cholistan were surveyed in the 1980s as part of an attempt to systematically survey the whole of Punjab (44). To date, Mughal’s publications constitute the largest and most detailed archaeological legacy data for this area, and the reported sites have been integrated into the corpus of Indus Civilization sites (45, 46). Through field walking and random surface collection, Mughal’s team noted 414 locations associated with different chronological periods ranging from the early Indus phases to the early Islamic. Petrie and Lynam (47) reviewed Mughal’s legacy data and incorporated sites reported by Stein in the 1940s, coming to a total of 462 archaeological site locations in the Cholistan region.

Some of the largest mounds discovered during the Mughal-led surveys have been the focus of recurrent visits by different teams (48, 49), particularly Ganweriwala, which traditionally has been considered one of the five major Indus cities together with Harappa, Mohenjo Daro, Rakhigarhi, and Dholavira (ref. 46 and Fig. 1 *A* and *D*), although its size and significance has been the focus of some discussion (48–51). Since Mughal’s surveys no major large-scale field surveys have been conducted in the region, despite the substantial and continued interest that these sites have aroused (52–57).

### Mughal’s Surveys: Site Distribution and Settlement Patterns.

**Fig. 2.**
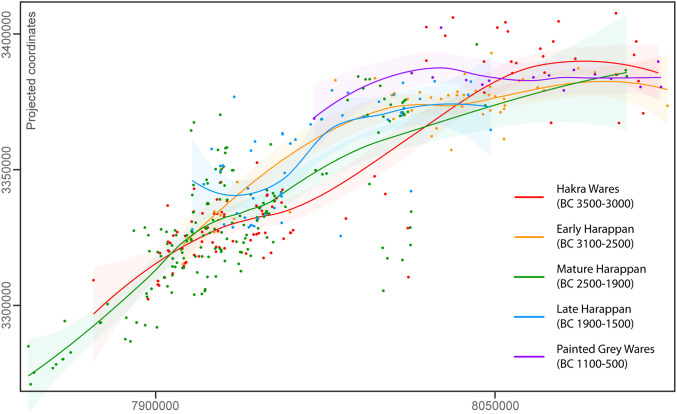
Spatial distribution of Mughal’s sites per period. Approximate distribution of previously known Indus sites in the area (see ref. 26, recently revised by ref. 47). The smooth regression lines represent the spatial trends in the distribution of sites. Although many of these coordinates need revisiting in the field, the chronological distribution of Mughal’s sites evidences continuous shifts in settlement.

**Fig. 3.**
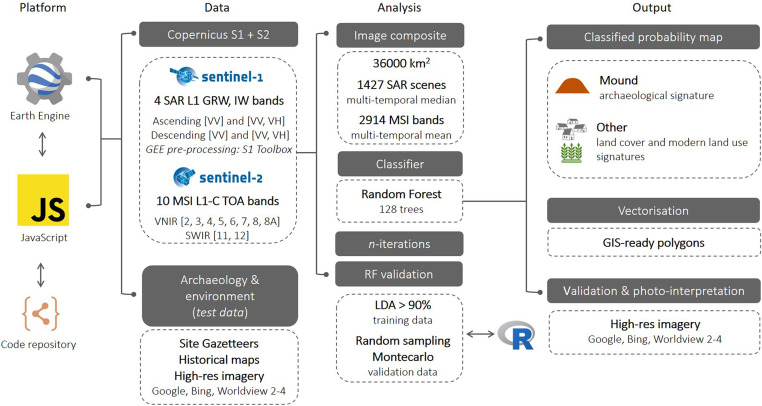
Schematic workflow used in this study. The code available within *SI Appendix* follows the three main steps of this research performed in GEE: 1) development of a multisensor, multitemporal image composite, 2) train and apply the RF classifier, and 3) export the resulting probability raster field. In addition, data validation and statistics were performed using R software.

**Fig. 4.**
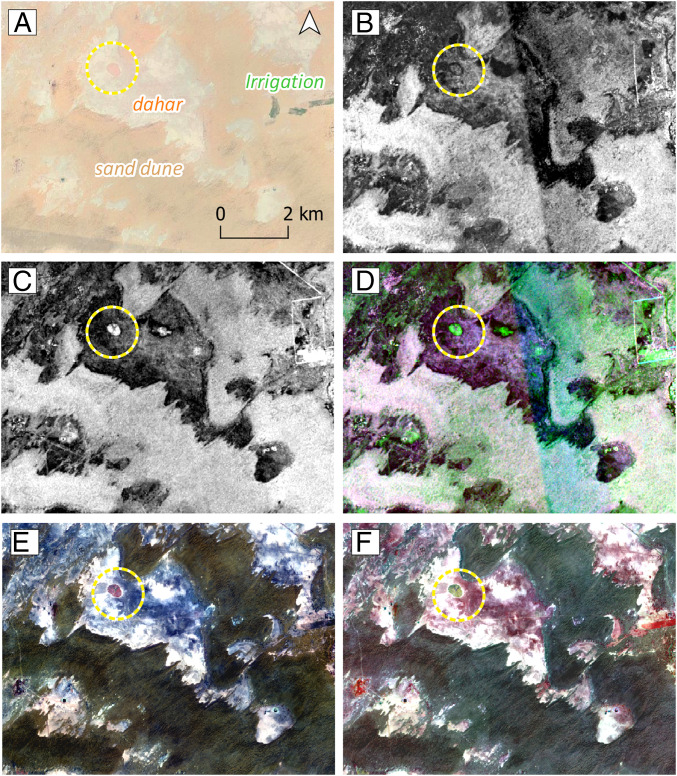
SAR and multispectral mound visibility. (*A*) Google Earth basemap showing the location of a well-preserved mound (yellow circle) and three main land-cover types in the desert edge: *dahars* or mud flat surfaces, stabilized sand dunes, and spots of irrigated lands. Note the differences in mound and land-cover visibility in the following band combinations from the multitemporal image composite: (*B*) dual Sentinel 1 band [VV,VH] in ascending mode; (*C*) single Sentinel 1 band [VV] in ascending mode; (*D*) Sentinel 1 false composite in RGB; (*E*) Sentinel 2 visible composite (B4-B3-B2); and (*F*) Sentinel 2 false color composite (B8-B4-B3).

**Fig. 5.**
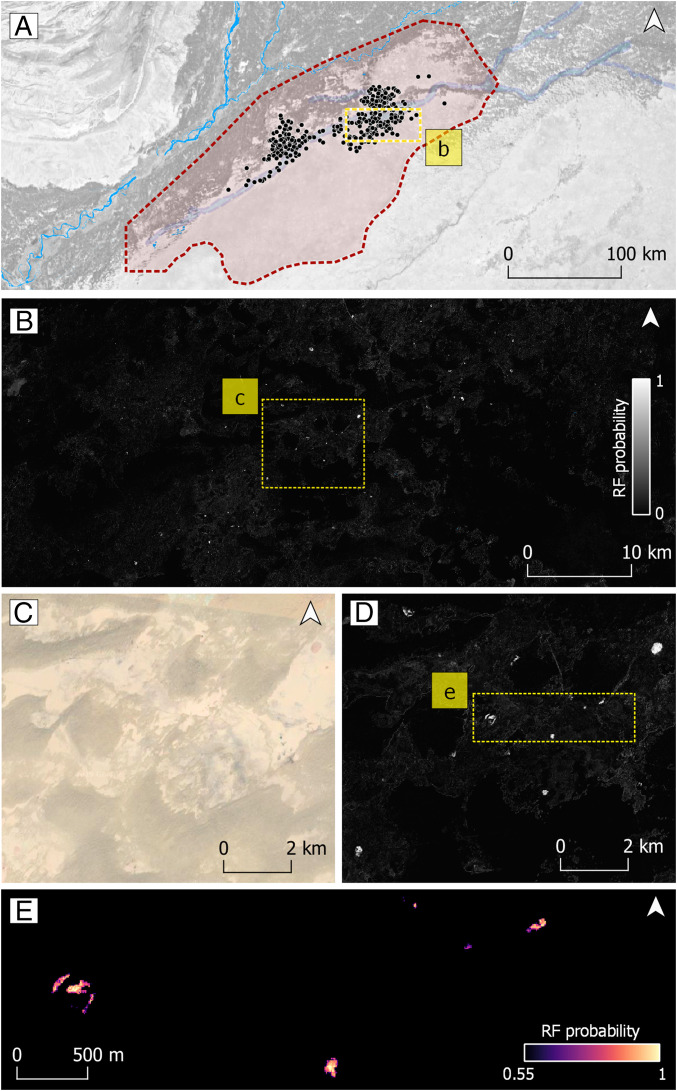
Results of the RF classifier. (*A*) In red, extent of the study area, showing the distribution of new RF probability mounds. (*B*) Inset showing the RF probability at the desert edge; note the white dots scattered through the region indicating high-probability mounds. (*C*) Visible high-resolution imagery (Google Earth basemap) with virtual absence of mounds. (*D*) The same area as *C*, showing high RF probability mound-like signatures in *dahar* surfaces. (*E*) Inset showing filtered pixels at >0.55 RF probability threshold, suggesting the presence of mounds partially covered by sand dunes.

**Fig. 6.**
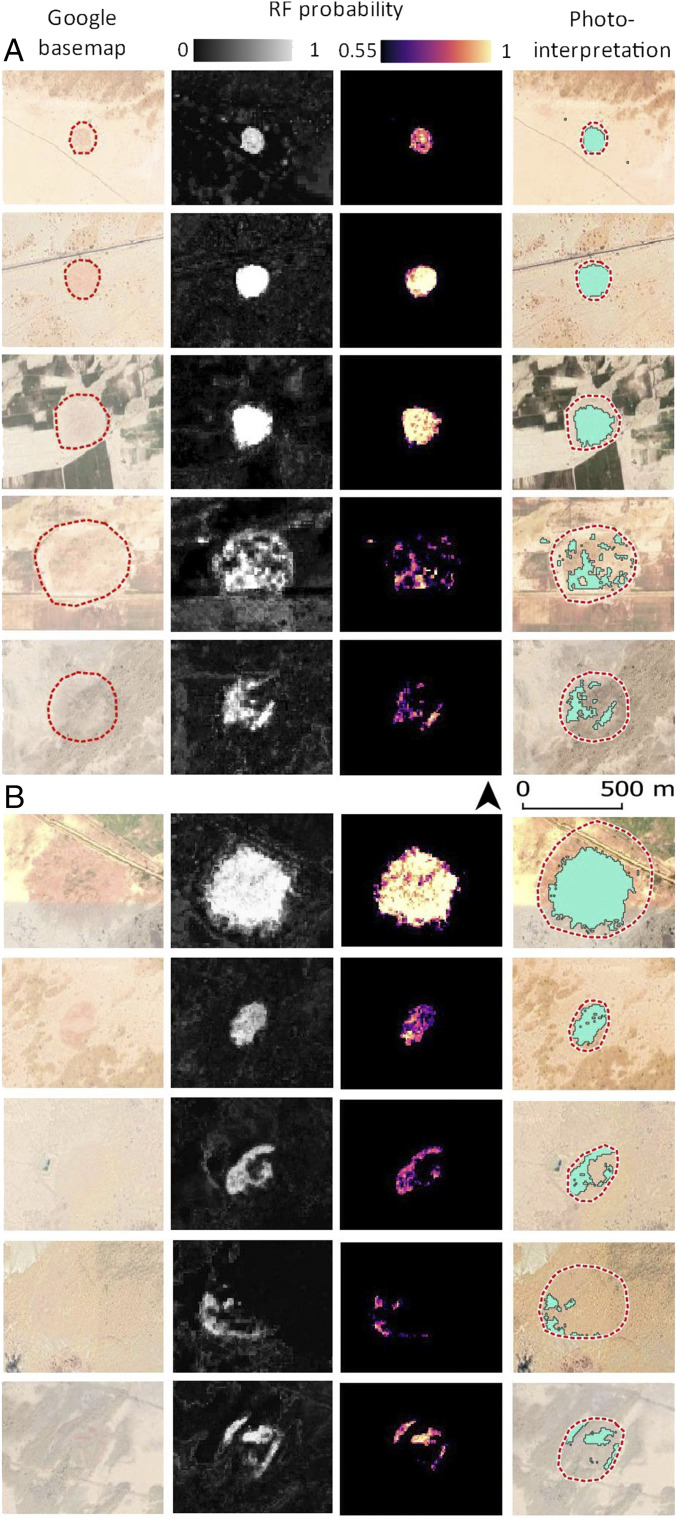
RF probability outputs. (*A*) Example of detected mound-like surfaces in well-known mounds used as validation set. (*B*) Clusters of high-probability pixels in the area, photointerpreted as new archaeological mounds. Note the distinct preservation of mounds due to partial coverage of sand dunes and desert shrubs.

Mughal found evidence for settlement distributions that varied across what he characterized as the Hakra, Early Harappan, Mature Harappan, and Late Harappan periods (26). The earliest, Hakra-period sites appear to be clustered to the south of Cholistan. There was a reduction of settlement in the south and displacement to the north in the Early Harappan period, a reduction of settlement in the north, and more extensive settlement in the south in the Mature Harappan period and abandonment of the south and a return to the north in the Later Harappan period. This alternating pattern is illustrated in Fig. 2. Mughal argued that these shifts in settlement concentrations were related to the movement of river channels and water availability (refs. 23–26, 57, 58 and Figs. 3–6). While the Hakra-, Early Harappan–, and Mature Harappan–period sites were distributed over the whole of the area, the Late Harappan–period sites appear to have been restricted to more northern locations. This displacement of settlement was not reversed in the subsequent Painted Gray Ware period, suggesting that during or at least after the Mature Harappan period the population made a major shift north.

### Old Limitations, Novel Approaches.

The Stein, Field, and Mughal data are inevitably constrained by the technological limitations of their time. The toponomy, location, and extension of mounded sites and scattered materials were recorded using manual and nonsystematic approaches, which makes it difficult to identify existing mound-site locations and to quantify their extension, exact distribution, and pattern (59, 60). A second problem is the lack of quantitative parameters that can help evaluating their significance. These data were usually published in the form of lists or gazetteers (e.g., refs. 26 and 46), lacking a quantified description of surface materials. Moreover, Mughal’s data (table 11 in ref. 26) include both mounded locations and other types of short-term occupation, such as industrial sites or campsites, and therefore many sites do not correspond to long-term mounded settlements. In addition, reported coordinates are often inaccurate and duplicated.

The lack of more systematic field surveys in recent decades is largely due to the remote location of most of these mound sites, the harsh desert conditions of the area, and its proximity to the India–Pakistan border. These limitations, along with the unique physiographic characteristics and archaeological significance of the region, make the Cholistan Desert an ideal scenario for testing new RS approaches.

## Materials and Methods

RS-based archaeological research in marginal or remote areas has often been limited by poor satellite coverage and limited temporal and spatial resolution. These limitations are changing thanks to 1) the availability of time series of global, medium-resolution satellite imagery from Earth Observation missions, such as the Landsat and Copernicus programs, and 2) the implementation of multipetabyte image catalogs and geospatial datasets in cloud computing environments that allow for planetary-scale analysis. For the analysis presented in this paper, the Copernicus Sentinel open access satellite series, in particular Sentinels 1 (both ascending and descending sensors) and 2, have been employed as they offer a higher resolution and greater number of bands than are available in other noncommercial satellite imagery. The original spatial resolution of the sensor bands employed, 10 and 20 m per pixel (m/px), is adequate for the detection of mound signatures in the area. These present a minimum diameter of around 100 m and, therefore, could incorporate enough number of pixels in sentinel imagery to be clearly identified. The Sentinel collections have been accessed and analyzed using Google Earth Engine (GEE) in order to investigate and automatically identify surface sediments potentially related to archaeological mounds. The methodological workflow is illustrated in Fig. 3. This combination of purposely built multisource multitemporal data and methods based on big data analysis has allowed the examination of a very-large-scale study area of *ca.* 36,000 km^2^.

### GEE Cloud Computing Geospatial Platform.

GEE is a fast-growing web-based geospatial platform seeing application within several academic disciplines, and in recent years it has boosted the emergence of RS-based automated applications at the continental and planetary scale of analysis (61). The archaeological application of GEE has been greatly extended only recently (12, 62–67). GEE is particularly suitable for implementing large-scale multitemporal data analysis as it provides access to a 20-petabyte catalog of satellite imagery and geospatial datasets, which includes the Sentinel series and most other publicly accessible satellite data acquired since the 1970s. GEE parallelizes and executes code developed in JavaScript or Python using Google’s cloud computing infrastructure, permitting work with intensive computational processes at unprecedented scales. GEE is also very useful for developing machine-learning processes as it allows the computation of partial machine learning-based classifications within a few seconds (or minutes if large training sets and many bands are employed) using screen map area and resolution. This is an important advantage to traditional machine learning processes as it allows the evaluation of the results of new training data without having to compute a full-resolution classification of an entire area and reduces the number of necessary iterations to achieve satisfactory results. GEE also incorporates high-resolution imagery (equal to that of Google Earth) that allows the evaluation of the results of the classification and the selection of new training data without needing to export them to GIS software. Furthermore, GEE provides vector drawing tools that simplify training data selection.

### Multisensor Sentinel Series.

Sentinel 1 is a dual-polarization C-band SAR with several scanning modes. The analysis presented here selected interferometric wide swath mode, which is the mainland operational mode with a ground resolution of 10 m/px and produces data in single (HH or VV) or double (HH + HV or VV + VH) polarization in both ascending and descending modes. Each scene available at GEE had been preprocessed using Sentinel-1 Toolbox to 1) remove low-intensity noise and invalid data on scene edges, 2) remove thermal noise, 3) radiometric calibration, and 4) terrain correction using SRTM 30 (spatial resolution of ∼30 m, 1 arcsecond at equator, absolute horizontal accuracy ≤20 m, absolute vertical accuracy ≤16 m, and relative vertical accuracy ≤10 m). Sentinel 1 provides data starting from October 2014.

Sentinel 2 multispectral imagery incorporates 13 bands from which only the visible/near-infrared bands (VNIR B2–B8A) and the short-wave infrared bands (SWIR B11–B12) were employed. Bands B1, B9, and B10 (60 m/px each) correspond to aerosols, water vapor, and cirrus, respectively, and they are not employed in this study except for the use of the cirrus-derived cloud mask applied. Visible (B2–B4) and NIR (B8) bands provide a ground resolution of 10 m/px, while red-edge (B5-B7 and B8A) and SWIR (B11–B12) bands present a 20 m/px spatial resolution. Specifically, for this research Sentinel 2 Level 1C products representing top of atmosphere (TOA) reflectance were preferred due to the larger span of its mission (starting from June 2015) and excellent resolution.

Machine learning-based approaches to the detection of archaeological sites have previously employed a single type of multispectral imagery source. This research has combined large multitemporal series of multispectral (Sentinel 2) and SAR (Sentinel 1) satellite data for the detection of archaeological mounds. Its use responds to SAR’s capacity to reflect soil roughness, texture, and dielectric properties (68) and other ground physical conditions such as compactness. The characteristic compact soil of archaeological mounds that have been formed by the decay of clay-based mud bricks was assumed to provide a stark contrast with surrounding desert soil (68–71). Another advantage of the use of SAR is that it has a certain amount of soil penetration in very dry, sandy, loose soils, which makes it particularly adequate for this specific area. Initial tests confirmed that known mound sites provided a characteristic SAR signature that differentiates them from the surrounding terrain (Fig. 4 *A*–*D*).

A notable drawback of single SAR images, which has been reduced here with the use of multitemporal series, is the presence of noise (speckle), an artifact of microwave scattering. Furthermore, SAR alone is not able to provide enough information to isolate archaeological mounds from other types of similar clayish soils (such as modern desert seasonal settlements and dried-up water *tobas*) that produce analogous responses. Equally, optical multispectral imagery is not single-handedly able to isolate mound spectral signatures in some other areas such those presenting natural accumulation of clays that produce similar spectral responses (Fig. 4 *E* and *F*). The different nature of these sensors was an important factor for ensuring that those elements that would produce values similar to those of mounds in one source were discriminated in the other. Given the complementarity of SAR and multispectral imaging, their combined use was conceived as a way of providing discriminant values for mounded archaeological sites in the area.

### Multitemporal Aggregates.

The use of multiple images makes it possible to consider short- and long-term environmental variability and different visibility conditions, thereby reducing the impact that incidental circumstances such as the presence of clouds have when using a single image. The only previous instance of the incorporation of multitemporal images for the machine learning-based detection of archaeological mounds has been by Menze and Ur (19). They used multiple ASTER satellite images of the same area acquired at different moments over a period of several years. Here, we have superseded that approach by employing a multitemporal fusion that averages 1,500 SAR images taken from 2014 to 2020 in the case of Sentinel 1 and 3,112 multispectral images acquired from 2015 to 2020 in the case of Sentinel 2.

An algorithm was developed (*SI Appendix*) to combine all available Sentinel 1 images for the study area and create a composite image integrating polarization and look angles to increase the amount of information available about the objects of interest. Median values were employed to integrate the different images available to ensure that we obtained a stable image and that radar speckle was eliminated. Medians were preferred to mean values to minimize the effect of eventual outliers.

While Sentinel 1 SAR is unaffected by clouds, Sentinel 2 TOA values may be affected by cloud cover. The S2 TOA collection reports coded information on quality concerns for each pixel in the QA60 bitmask. We integrated all multitemporal images of the Sentinel 2 collection into a single multispectral image by averaging the pixel values per each band but ignoring those observations flagged as cloudy (opaque or cirrus) in the QA60 bitmask.

Sentinel 1 and 2 data aggregates made it possible to produce a 14-band multitemporal and multisensor image composite. The image integrates 4 SAR bands (a single VV and a dual HH–HV band in both ascending and descending modes) and 10 optical multispectral bands (B2–B8A, B11, and B12). The high-quality optical and radar bands are not affected by particular environmental or visibility conditions and therefore reflect average reflectance values for the study area while significantly reducing the computational costs of the process. While incorporating seasonal information might have produced improved results (see, e.g., refs. 64 and 72), we preferred to test the most straightforward approach of using aggregate averages, thus keeping computational cost relatively low. This is a particularly important point as the algorithm employs two sensors and a relatively high spatial resolution given the very large size of the study area.

The creation of multitemporal and multisensor (active and passive) aggregates for the detection of archaeological sites constitutes an important development. Previous research has emphasized the current need for “the harmonization and synergistic use of different sensors … to maximize the impact of earth observation sensors and enhance their benefit to the scientific community” (73). In this regard, this research constitutes one of the first large-scale applications of the concept of virtual constellations, a “set of space and ground segment capabilities that operate in a coordinated manner to meet a combined and common set of Earth Observation requirements” put forward by the Committee on Earth Observation Satellites (ref. 74; see also ref. 75) and falls in line with current agendas for the advancement of archaeological RS (76, 77). Virtual constellations aim to combine sensors with similar attributes to increase the efficiency of RS processes. Here, we have gone beyond the initial definition to include sensors with very different principles (active and passive), but with the combined potential to produce superior results given the nature of our object of interest. The use of a machine-learning algorithm provided a practical way to employ the multiplicity and disparity of data present in the image composite bands.

### Machine-Learning Algorithm.

The steps for classification of mound-like signatures included gathering training data, training the classifier model, classifying the image composite, and then validating the classifier with an independent validation set. We employ a selection of 25 mound sites identified in Mughal’s survey on desert areas (26) as our training (*n* = 5) and validation (*n* = 20) sets. Despite the quality problems in Mughal’s data, we selected those sites that could be clearly identified and accurately located in high-resolution imagery available in GEE. These corresponded to large and well-preserved sites. Polygons were drawn in GEE to define the areas of the selected mounds from which the values of the pixels in the image composite could be extracted for the training of the algorithm. The definition of spectral signatures and the evaluation of training data are described in detail in *SI Appendix*, Figs. S1 and S2.

An RF classifier was selected for the GEE machine-learning implementation. A RF classifier builds a number of decision trees on bootstrapped training samples, but each time it considers a split in a tree. For each split, a new random subset of predictors is considered when splitting nodes (78). The average of the resulting trees helps to avoid overfitting and hence is less variable and more reliable than other decision tree-based classifiers (17, 79). Moreover, RF classifiers can handle a small number of training samples and it is possible to get the number of votes (i.e., the probability density) for each class. These are two advantages that are particularly useful for RS-based archaeology with limited land-use/cover information.

In our GEE algorithm, the RF was composed of 128 trees, considered an adequate number to obtain optimal results without increasing computational cost unnecessarily (80). The RF was set in probability mode so that the results could be evaluated, filtered, and refined to improve the algorithm’s detection capabilities. The machine-learning process underwent three iterations. The original iteration of the algorithm produced satisfactory results in that it was possible to clearly identify the 20 well-known mounds used as test data and many more potential mounds through their higher RF probability values. Nonetheless, two more iterations were necessary to tune pixels with higher percentages that did not correspond to mound sites, thus ensuring a good balance between low presence of nonmound pixels with high RF probability values and a high mound detection rate. The output of the RF classifier is a probability field in raster format, in which each value records the probability of a given pixel being a “mound.”

In order to produce a map of archaeological mounds, a >0.55 RF probability threshold for mound values was selected after close inspection of the training data on the high-resolution imagery, which produced a raster map of clusters (“mounds”) on a background of “no mound.” A higher threshold resulted in the better delineation of big and clear mounds, but many small clusters of pixels corresponding to partially covered or small mounds were lost. We considered the >0.55 threshold a good compromise between a high mound detection capacity and a minimal inclusion of false-positive pixels (mainly scattered, isolated pixels). The algorithm’s validation and quality assessment methods are outlined in *SI Appendix*, Figs. S3 and S4.

### Integration of Complementary Data, Area Estimates, and GIS Database.

The resulting clusters of high-RF-probability pixels representing mounds were vectorized to reconstruct the areas of the mounds currently covered by sand dunes or desert shrubs. Photointerpretation used high-resolution satellite imagery provided by several map services (including Google Earth and Bing Maps) and a limited collection of available WorldView-2 and -3 imagery. The combination of these sources provided enough temporal and environmental variability to evaluate and delineate the possible extent of the mounds identified by the algorithm. A final mound geodatabase was prepared in a GIS environment and compared with previous coordinates from legacy data (see refs. 26 and 44).

### Data Availability.

All satellite data used in this study are freely available under the open data policy adopted by the Copernicus program of the European Space Agency. The code developed for this research, which has been used to produce the results discussed in the paper, is provided within *SI Appendix*. This code has been written for GEE’s implementation of JavaScript. GEE provides free access to the satellite data and the processing necessary to conduct the analysis upon registration. The code is ready for the direct execution of the analysis discussed in this paper (including the gathering and treatment of Sentinel data) and it only requires pasting into the GEE Code Editor and pressing the “run” button. It includes instructions on how to use and modify it to be applied to other research needs or areas. The code is also available in an online repository at https://github.com/horengo/Orengo_et_al_2020_PNAS, where future updates will be implemented.

## Results

### RF Probability Field.

Thresholding the RF probability field at >0.55 resulted in a map of 337 clusters that we propose as archaeological mound soil surfaces (Fig. 5 *A*–*E*). This set includes the 25 mounds selected from Mughal’s surveys (26) used as training (*n* = 5) and validation (*n* = 20) data, which were all successfully identified by the algorithm. The newly proposed mounds are similar to the previously known mounds used as training data (Fig. 6*A*). The capacity of the algorithm to detect mound-like signatures is probably related to 1) the high contrast in Sentinel 1 single and dual polarization bands (81), 2) the ability of the SAR C-band to penetrate loose, dry sand (82–84), and 3) the specific reflectance in the Sentinel 2 red-edge, NIR, and SWIR bands of anthropic sediments in mounded sites (85, 86).

Due to vegetation and sand cover the RF probability only produced a few well-defined rounded shapes. Most of the newly proposed mounds present fragmentary rounded shapes, elongated strips, or shapeless groupings of pixels (Fig. 6*B*). It is entirely possible that beside these 337 detected mounds there are also remains of archaeological mounds partially covered by sand or shrub vegetation with low RF probability values. It is worth stressing that the algorithm helped to identify small clusters of mound-like pixels even when visible high-resolution images or SAR backscatter alone do not show any significant change in surface land cover.

When possible, the new features were matched with legacy data from previous archaeological sites recorded by Mughal’s team (26, 44). Out of a total of 337 clusters of high-probability pixels identified as archaeological mounds by the algorithm, only 71 (including the 25 employed to train and test the algorithm) could be linked with reasonable certainty to sites previously recorded. However, it is possible that some other mounds in the database were indeed recorded by Mughal, but their imprecise coordinates make it difficult to establish any secure spatial correlation.

## Discussion

### Detection and Distribution of Mound-Like Signatures.

**Fig. 7.**
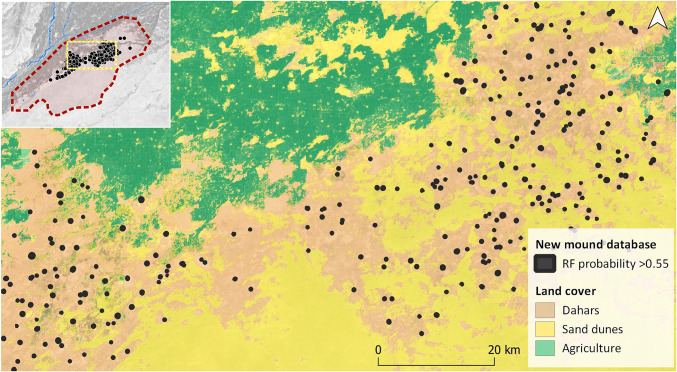
Distribution of newly detected mounds in relation to regional landcover.

Despite the intensity of the regional surveys conducted by Mughal’s teams in the 1970s and 1980s, the automated detection of mounds in Cholistan has significantly increased the number of mound-like settlements over a much larger area of Cholistan than previously thought. In particular, the distribution extends toward the southern part of the region and the inner Thar Desert toward the east, which was an area that was virtually inaccessible for previous studies (Fig. 7).

As expected, the distribution of the detected mounds by the algorithm was concentrated in the desert areas. Multispectral contrast and SAR texture differences among classes are accentuated in the desert, especially in the *dahar* flat surfaces, whereas the environmental variability in the agricultural lands toward the western edge of the desert is highly affected by several factors (e.g., mechanized agriculture, irrigation canals, roads, and railways, and irrigation-supported agricultural villages). As a result, the visibility of most existing archaeological mounds in this area has been strongly influenced by present-day taphonomy.

There is also a clear decrease in site density toward the middle of the overall site distribution (Fig. 7). This can be attributed to an extended stretch of deeper dunes that potentially hides a similar site density to those documented west and north of the dune stretch. In a similar manner, the area where new mounds have been identified is delimited to the south and southeast by the presence of deep dunes. The distribution of documented sites in relation to the presence of dunes (Fig. 7) strongly suggests that many more sites could be lying below dunes in the deeper desert, and therefore the depositional dynamics of aeolian sediments in the Cholistan Desert may have played a crucial role in both the settlement history of the region and visibility of mounds. It is also interesting to note that the areas with a higher concentration of sites are situated in open mud flats (*dahars*) that are scattered through the region, although many of these mounds are still partially covered by sand dunes. Some *dahars* are used today to extract fine silty sediments or to excavate *tobas* (25, 27). These activities have accumulated silty soils on the *dahar* surface, leaving soil signatures and texture that are very similar to those of mounds in single Sentinel 1 and 2 scenes and high-resolution imagery. In all cases, the RF probability field was able to clearly discriminate between *dahars*, *tobas*, and archaeological mounded areas.

Only 71 of the mounds located by Mughal match the new photointerpreted RF probability mounds, and usually these are among the most well-preserved mounds in the landscape. A large number of nomadic sites and pottery distributions not related to long-term settlement were reported by Mughal, but they were only distinguished on site by surface scatters of artifacts (27), and the resolution of our sources is not sufficient to locate them. An important aspect of the RF probability approach is that the new locations represent areas with a specific mound-like sediment signature, which suggests a relatively stable occupation, potentially for some time, and the continued use of construction materials such as mud brick. These characteristics are closely associated with sedentary dwellings in similar Indus contexts (51).

**Fig. 8.**
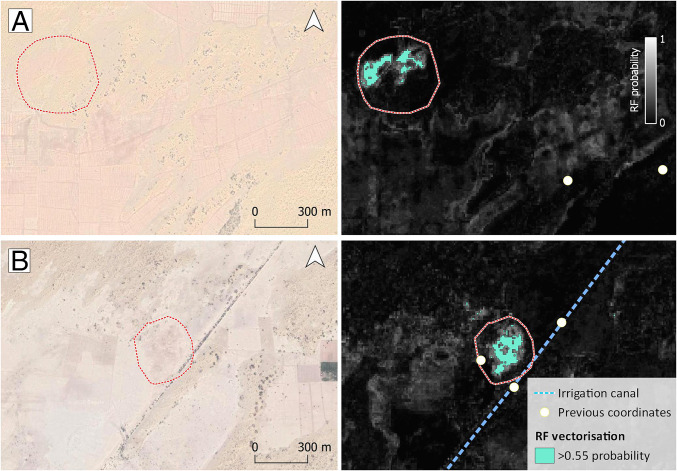
Visibility of RF mounds and legacy data. Google basemaps and RF probability fields showing (*A*) the vectorized new mound of Bokhariyanwala, closely located to multiple legacy coordinates for the same site, and (*B*) the vectorized new mound of Changalawa, also reported as multiple locations in legacy data probably due to the partial obliteration of the site by an irrigation canal.

For each new location, the extension, visibility, and preservation of surface archaeological sediments can be now further explored in relation to its immediate surroundings. Previous studies tended to separate a unique multiphase location into several distinct locations based on the distribution of surface material culture, which are largely biased by the terrain view that was used (no aerial images were available) and the partial burying and occlusion of sites. For example, the site of Bokhariyanwala was described as having occupation during the Mature and Late Harappan periods on two adjacent mounds. Our analysis shows that the ridge of a fossilized dune crosses the area, suggesting that these sites formed a single large mound (Fig. 8*A*). Similarly, the site of Changalawa was reported to have two distinct Mature Harappan–period mounds and a third Late Harappan–period mound. However, the RF-probability field only returns a single mound in this area. In this case, the distribution of materials may have been disturbed by the presence of a 1920s irrigation canal crossing the site, and different parts of the one site may have been occupied in each period (Fig. 8*B*). However, until archaeological excavation can be carried out at these sites, the possibility that some of these sites may be composed of discontinuous occupations on multiple overlapping mounds cannot be dismissed. These examples reflect the high heterogeneity in terms of site visibility and preservation across the study area and provide a cautionary tale on using the previous data to study site density, distribution, and size.

### Villages and Towns: Mound Size Estimates.

**Fig. 9.**
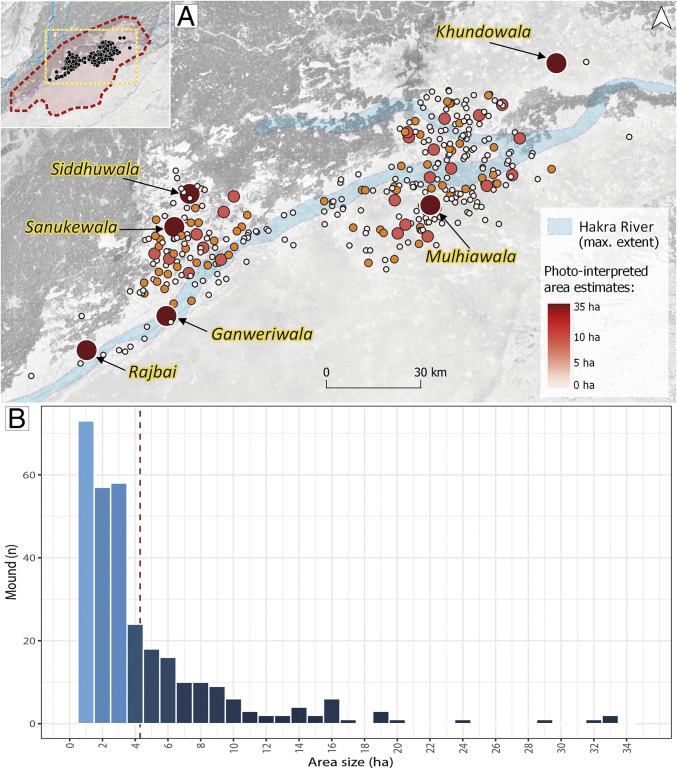
Mound area estimates. (*A*) Map showing the mound size estimates. The location of Ganweriwala and the other five large mounds (>20 ha) located in the region is highlighted. Note the extension of the Ghaggar-Hakra River, as digitized from historical maps. (*B*) Histogram with the size density for the mounds. Mean size is indicated by the red dashed line.

An important result of the automated site detection has been the delineation of the estimated area size for the 337 mounds located in the area (Fig. 9 *A* and *B*). Mound area estimates in the Near East (87) have raised the question of whether these represent the total habitation size of a settlement or wall debris and open-air spaces outside the settlement. For the results presented here, area estimates should be used with caution and be considered only as a tentative measure of the total mound area estimated through visual inspection of the RF probability field and high-resolution satellite imagery. A large proportion of the newly detected mounds are less than 5 ha in size (*n* = 246, 72.99%). Estimated site areas suggest a general pattern of small rural Indus settlements distributed between medium- and large-sized sites that are possibly urban in nature (26, 88), which is similar to other Indus core areas in northwestern India such as Gujarat (89) and Haryana (90). However, the area for most of the known sites that we have identified is slightly different from those previously reported (table 13 in ref. 26). For example, the Hakra-period site of Lathwala was reported to be 26.3 ha, but the closest visible mound is around 5 ha, although it should be noted that the site is now clearly divided by a large sand ridge. The largest Early Harappan–period site, Gamanwala, was reported as being 27.3 ha, but the closest mound to this location is a much smaller mound of *ca.* 6.5 ha. Kudwala, the largest Late Harappan–period mound at 38.1 ha, could not be detected remotely, suggesting either that its original location was not correct or that it has since been obliterated by the expansion of agricultural land. Significantly, the RF-probability field detected six mounds that can undoubtedly be considered as large Indus settlements, potentially towns of more than 20 ha. The distribution of these mounds is highlighted in Fig. 9 *A* and *B*. The Mature Harappan–period settlement of Ganweriwala, initially estimated to be 80 ha in size (26), has long been considered one of the major centers of the Indus Civilization. However, recent reassessment has shown that it was much smaller, between 20 and 40 ha (49–51). Our data suggest that the mounded area is *ca.* 33 ha, comparable to a large town (90), although it may well have functioned as an urban center (51). It is possible, however, that Ganweriwala was not the only large town dating to the Mature Harappan period in the region. Two more sites identified by Mughal as dating to this period, Sanukewala and Rajbai, have a similar visible mound extension (*ca*. 32 ha) and they could have been even larger in the past. The northwest section of Sanukewala is largely affected by an abandoned canal and Rajbai is partially covered by sand dunes. The Late Harappan–period site of Siddhuwala also has significant proportions (26.5 ha). The RF-probability field has also brought to light two previously unknown large sites, named Khundowala (29 ha) and Mulhiawala (20.5 ha) after the nearest toponym shown on historical maps. Mulhiawala is in the southeastern margin of the desert, an area that was virtually empty of Indus sites before the automated detection of mounds. The site of Khundowala is unique in lying in the northeast margin of the desert, and it is completely isolated from other visible mounds. The site is partially covered by a fossilized sand dune, and indeed the RF probability only showed a small portion of the total mound that can be appreciated today in high-resolution satellite imagery. The identification of this site is highly relevant, as it documents the presence of a large mound in the northern parts of the historical Hakra River basin. Despite previous evidence of the presence of more Indus Civilization sites in this area (26, 44), only a few small mounds have been detected by the algorithm there. The implications of the northern expansion of the desert, as evidenced by the large sand dunes that partially cover these sites, is further discussed below.

### Long-Term Landscape Dynamics.

The new distribution of mounds in the study area indicates that the Thar Desert has expanded considerably since the Indus period. No current occupation except for seasonal pastoral camps is known in the southern and western sectors of the site distribution area. Medieval- and late Medieval-period settlements such as the Cholistan forts known in the area form an arc delimiting the northwestern limit of the Thar Desert at a more northerly and westerly location (26). These patterns might suggest that the expansion of the desert is a long-term process that has been progressing over several millennia, contributing to the abandonment of settlements at the desert margins.

At present, the lack of paleoenvironmental evidence such as sedimentary records in the core area of the Cholistan Desert can only be partially complemented by data from the adjacent Thar Desert, especially in western and northern Rajasthan. Durcan et al. (91) have suggested that the Holocene geomorphological dynamics were highly dynamic and the distinct phases of fluvial and aeolian deposition were not spatially or temporally instantaneous, but rather a synchronic alternation. Some studies have, however, highlighted a drier climatic condition on the northern margins of the Thar Desert sometime after ∼4.4 ± 0.1 ky B.P. (24, 59, 92–94), a phase that is consistent in other northwestern regions such as mainland Gujarat (95, 96). Nonetheless, the cyclic fashion of the Quaternary aeolian activities in the Thar Desert started much earlier (*ca*. 150 ky B.P.), and Singhvi and Kar (97) advocate for a phase of major aeolian activities during the Holocene Climatic Optimum (*ca*. 5 to 3.5 ky B.P.), with century-scale rates of spatial shifts in dune forming in west Rajasthan up to 2 ky B.P. Existing sedimentary records for the western margins of the Thar Desert, therefore, correlate well with a potential enhanced aeolian activity *ca.* 4 ky B.P., followed by declining rainfall, which, in turn, would have stabilized the now subdued sand dunes. Another burst of aeolian activity started *ca.* 2 ka while the latest aeolian active phase was in historical times, when the rates of dune mobility in the desert increased mainly due to human pressure (98, 99).

The new data on mound distribution can also be compared to the documentation of multiple paleoriver channels and seasonal streams in the study area. Just as with the mounds, these are visible only in *dahar* surfaces and therefore it is difficult to offer a continuous picture of their shape and length. Overall, the hydrological network in the area has been related to the course of the former Ghaggar-Hakra River. Some authors have argued that the Ghaggar-Hakra system ceased perennial flow through this region before the Holocene (59, 100–103), thus suggesting that the alluvial plains of northwestern India and eastern Pakistan were characterized by a continuum of fluvial environments interrelated through a seasonal precipitation gradient and local aeolian dynamics (36, 104). The river basin is well-documented in historical 19th-century maps and narratives (Fig. 9), showing recurring seasonal flooding during episodes of extreme rainfall such as in 1804, 1805, and 1871 (105). However, previous RS-based attempts to identify the former channel network across Cholistan have suggested several potential interconnected waterflows (106–109), but little is known about their chronology. Moreover, the detected relict network of fluvial environments across the region has several characteristics that differ from a major Ghaggar-Hakra course, specifically 1) these paleochannels form multiple courses with roughly similar orientation, 2) their morphology is relatively straight, at least by comparison with those documented to the north of the study area, where they tend to be very sinuous given the low slope of the alluvial plain (see refs. 64, 65, and 110), and 3) their orientation is coincident with that of the fossilized dunes and not with that of the alluvial plains just north of the desert edge. In this regard, it is important to note that while river flow follows the aspect of the terrain, dunes are mostly influenced by wind direction in plain areas. Therefore, the flow of many of the rivers detected must have been influenced by the presence of earlier relict sand dunes. The many subparallel, ephemeral river traces in the area might be related to a process of river migration toward the northeastern region as dunes expanded from the south. Consequently, it is possible to propose that water was still available, at least seasonally, in streams and flooded fertile *dahars* until recent historical times, as attested by the nomadic migration traditions in the area (27, 111). The monsoonal-fed hydrological network changed dramatically from 1897 onward with the construction of the Ottu Barrage on the northern Indian course of the Ghaggar River, which increased desertification in the southern desert edges and led to the development of large irrigation schemes through the desert lands early in the 1930s (112).

### Indus Settlement Trends.

Although at the moment it is not possible to provide chronological information for the newly detected mounds, they can be combined with those previously identified and dated by Mughal (26) to attempt to understand changes in settlement distribution over time. The surveys of Cholistan documented all of the mounds that were encountered, including those of the Medieval period. Considering that the vast majority of the previously reported sites were settlements attributed to the periods of the Indus Civilization, there is a high chance that most of the newly detected mounds were also occupied during this same period. This assumption can be contrasted with similar Indus contexts. For example, in the plains of Haryana in northwest India, Green et al. (14) have highlighted that mound features visible on historical maps tend to be protohistoric or Bronze Age settlements when surveyed or validated on the ground, whereas Early Historic and Medieval settlements appear to be more frequently associated with modern settlement locations. In Cholistan, specifically, several mounds are partially covered by fossilized sand dunes and ridges, indicating that these mounds largely predate the northwestern expansion of the Thar Desert, and that mounds located deeper in the desert may therefore be particularly early in date.

Mughal’s analysis highlighted the importance of the Cholistan settlement data for understanding the development of Indus urbanism, and Madella (113) has suggested that Cholistan was potentially a zone of intensive and extensive cultivation. Taken at face value, Mughal’s settlement distribution data for Cholistan suggest that this was an intensively occupied area. However, Petrie and Lynam’s (47) reanalysis of the data suggests that this settlement system may have been marked by displacement and considerable instability, which indicates that it was more unusual than is typically assumed. This interpretation has ramifications for the interpretation of agricultural practices and the sustainability of the region, indicating a high degree of flexibility and mobility. This insight is important, because there is currently no direct archaeobotanical evidence available from sites in this region. This agricultural flexibility must have been essential to Cholistan, which was strategically located in a central point of the area occupied by Indus populations (51, 114) and was potentially a necessary transit area for movement between different regions. Cholistan may well have formed a communication node between surrounding Indus areas. The apparent loss of the more southern areas of Cholistan for settlement may have been an important factor in the breakdown of Indus interaction networks and the increase in more local-scale interactions in the Late Harappan period (115). The decline of settlement in Cholistan might thus have created a “dead zone” of interaction, increasing the cost of communication and exchange beyond the point that deurbanizing cities could maintain.

## Conclusion

The dataset provides a collection of Sentinel 1 and Sentinel 2 spectral signatures for mound-like archaeological features in drylands, and the resulting mound locations can be now addressed in terms of RF probability values. We present a combination of multitemporal, multipolarization, and multiangle SAR bands and multitemporal optical bands (including visible, red-edge, NIR, and SWIR) analyzed using a machine-learning algorithm in a cloud computing platform for the detection and analysis of archaeological mounds, which has the potential to transform archaeological site detection. The machine-learning algorithm that has been employed was able to detect all previously known mounds in the study area for which we could gather accurate locations and large numbers of new ones well beyond the expectations laid out by previous research. The method provides results that are noticeably superior to the use of single-source RS approaches. RS-based applications in arid and semiarid areas elsewhere can benefit from the integration of globally available Sentinel data in GEE’s accessible, flexible, and reproducible environment to perform and evaluate machine-learning workflows.

The new distribution of archaeological sites in the Cholistan Desert, in combination with legacy archaeological data, suggests that most of these mounds are protohistoric settlements, that they extended across a larger area than previously recognized, and that they include several previously unknown mounds that, considering their large size, can be classified as urban in their own right. Archaeological data in combination with landscape analysis, which includes the mapping of factors affecting site visibility, suggest that these were only a relatively small part of the mounds present in the area, many of which might lie below large dunes in the core study area and deeper into the desert.

The archaeological sites in Cholistan were occupied at points along a span of five millennia. The significant number of Indus Civilization Mature Harappan–period and urban-scale mounds that were documented and the shift in the concentrations of settlement to the north suggests that it is important to consider the impact of the advancement of the desert on settlement displacement of Indus populations. Given the centrality of this area, the displacement of occupation in Cholistan potentially played an important role in the generalized processes of deurbanization, decrease in settlement size, and regionalization that characterize the Late Harappan period across the Indus region.

While undoubtedly Cholistan was a significant zone of settlement for the populations of the Indus Civilization, the nature of the settlement dynamics in this region and their relationship to water availability are in need of both ongoing reevaluation and ground-truthing in the field. Indus populations had clearly adapted their behavior to survive in this apparently unstable environment, and it appears to have remained an important area for settlement and a component in interactive networks for an extended period. Future research should perhaps also consider the role of pastoralism and the pastoral economy in this region and its possible links to population mobility. The results of the study presented here provide critical resources for the processes of rethinking the dynamics of settlement distribution and the archaeological significance of the region.

## Supplementary Material

Supplementary File
