# Supplementary material for: Automated detection of archaeological mounds using machine-learning classification of multisensor and multitemporal satellite data
Source: Proc Natl Acad Sci U S A. 2020 Jul 20;117(31):18240–50. doi: 10.1073/pnas.2005583117 (PMC7414161; doi:10.1073/pnas.2005583117)
Supplement: Supplementary File [file pnas.2005583117.sapp.pdf]

PNAS
www.pnas.org

Automated detection of archaeological mounds using machine learning classification of multi-sensor and multi-temporal satellite data

H.A. Orengo*, F.C. Conesa*, A. Garcia-Molsosa, A. Lobo, A.S. Green, M. Madella and C. A. Petrie

*H.A. Orengo
E-mail: horengo@icac.cat
*F.C. Conesa
E-mail: fconesa@icac.cat

This PDF file includes:

Text providing full details of methods (i.e. validation of training set and machine learning algorithm), with the following subsections:

Preliminary assessment: image composite and spectral signatures
Linear Discriminant Analysis
Quality of the RF probability field

Figures S1 to S4.
References for this SI file

JavaScript GEE code of the algorithm described and employed in the paper. The code also incorporates detailed explanations of each step followed and instruction on how to modify the algorithm to be applied in other study areas. Future versions and improvements of this code will also be made available in the following link:

https://github.com/horengo/Orengo_et_al_2020_PNAS

Note: the font size of the code has been reduced to avoid line breaks, which could affect the execution of the code when copied and pasted elsewhere. Also, the transformation of this file into a PDF could affect line breaks and make the code unusable. Please, refer to the website above for a working version of the code.

www.pnas.org/cgi/doi/10.1073/pnas.2005583117

Full details of methods: training data and validation of machine learning algorithm

Preliminary assessment: image composite and spectral signatures

In order to evaluate the distinctiveness of the mounds in terms of optical and SAR values, the multispectral and SAR signatures from our training set (i.e. “mound” pixels, n= 3360) were compared to an additional dataset of two well-defined land cover types in the region that did not correspond to mound-sites: “desert” pixels, including *dahar* surfaces and sand dunes (n= 11055), and “irrigated” pixels, mostly representing cultivated or vegetated fields (n= 5860). In addition, those signatures were also compared to a random set of 5000 points/pixels distributed within the study area.

The 14-band image composite provided satisfactory results for the preliminary assessment of the quality of the training data. In general terms, pixels classified as “mound” in the training set produced a specific spectral reflectance in all of the Sentinel 2 bands, which greatly differs from the spectral behaviour of “desert” and “vegetation” land-cover types (**Figure S1**). In particular, the spectral variability of mounded-sites is lower than other classes in S2 bands B2 (Blue) and B3 (Green). In contrast, its reflectance is higher in S2 bands B4 (red) and B5 (red edge). As expected, reflectance of training pixels classified as vegetation is higher towards the NIR edge of the spectrum (red edge B6 and B7, and NIR B8 and B8A), whereas the spectral behaviour of vegetation is lower in SWIR bands (B11 and B12). Interestingly, pixels from well-preserved mounds can be easily distinguished from the surrounding “desert” areas characterised by sand dunes. In contrast, values from Sentinel 1 shows a much more homogeneous SAR response to target training areas in both ascending and descending scan orbits. Nonetheless, a clear difference in the radar backscatter (measured in dB) is observed between the Sentinel 1 single polarisation bands [VV] and the dual polarisation bands [VV,VH]. This abrupt contrast between SAR bands might be responsible for a more accurate detection -by means of SAR contrast and soil roughness- between distinct surface targets such as archaeological mounds.

Linear Discriminant Analysis

Furthermore, a Linear Discriminant Analysis (LDA) was performed using R software (1) and its package MASS (2) to inspect the relative positions of the different training pixels (i.e. “mound”, “desert” and “irrigated”) on a reduced space. LDA produces a linear transformation in which the classes are optimally linearly separated in terms of within-class vs. among-class covariance (see 3, 4). The additional random set of pixels was also projected on the LDA space to further inspect the distinctiveness of the mounds.

Figure S2 shows the LDA canonical plane with the first two LD components, indicating clear spectral differences among the predefined training classes despite the within-class variability. “Mound” pixels are clustered towards the left side of the plane, whereas “desert” pixels and “irrigated” pixels are clustered in the upper and lower central plane, respectively. The distribution of the set of random pixels in the LDA plane indicates that most of the spectral variability in the region spans from “desert” to “irrigated” land cover types, which includes *dahar* surfaces, relict sand dunes and healthy vegetation in cultivated fields. This distribution explains the distinctiveness of “mound” pixels in the study area.

Quality of the RF probability field

To assess the quality of the RF probability field we calculated the distribution of RF probability (i.e. percent of pixels by RF probability intervals) for those pixels included within the validation set of 20 mounds and compared to the null hypothesis of a random distribution of the spatial polygons. For the purpose of estimating the distribution of RF probability under the null hypothesis of no association of RF probability to actual mound occurrence, the 20 polygons were randomly distributed within the area of study and the distribution of RF probability was re-calculated as for the actual sites, repeating this process 1000 times.

The distribution of RF probability within the 20 digitised well-known mounds used as validation set was significantly different to that of the 20 randomly located polygons (**Figure S3a-b**): 20% of pixels within digitised mounds had low RF probability values (0 - 0.05), and 7% had high RF probability values (0.95 - 1), while randomised polygons had 95% of pixels with low RF probability and only 0.01% with high probability. We assessed the usefulness of the RF probability field by calculating the density (d(i)) of validation mound pixels at a given RF probability value, i.e. for each interval i of RF probability, d(i) = nb

of mound pixels(i)*100/nb pixels(i)]. Interestingly, the barplot of density of validation mound pixels (**Figure S3c**) is heavily skewed towards intervals of high RF probability, indicating much higher probability of finding a validation mound in the areas with high RF probability and encouraging that screening areas of high RF probability should result on a very efficient method for locating mounds.

Finally, we repeated the assessment of the density of mound pixels by RF probability intervals with the final mound database (i.e. the 337 photo-interpreted mound polygons), as done with the validation polygons. **Figure S4** shows the density of the Cholistan sites at any given RF probability intervals. The higher density of archaeological mounds is well beyond the >0.55 RF probability threshold, thus indicating a robust confidence in the algorithm capabilities to detect soil and sediment signatures related to potential archaeological mounds in desert areas.

Supplementary figures

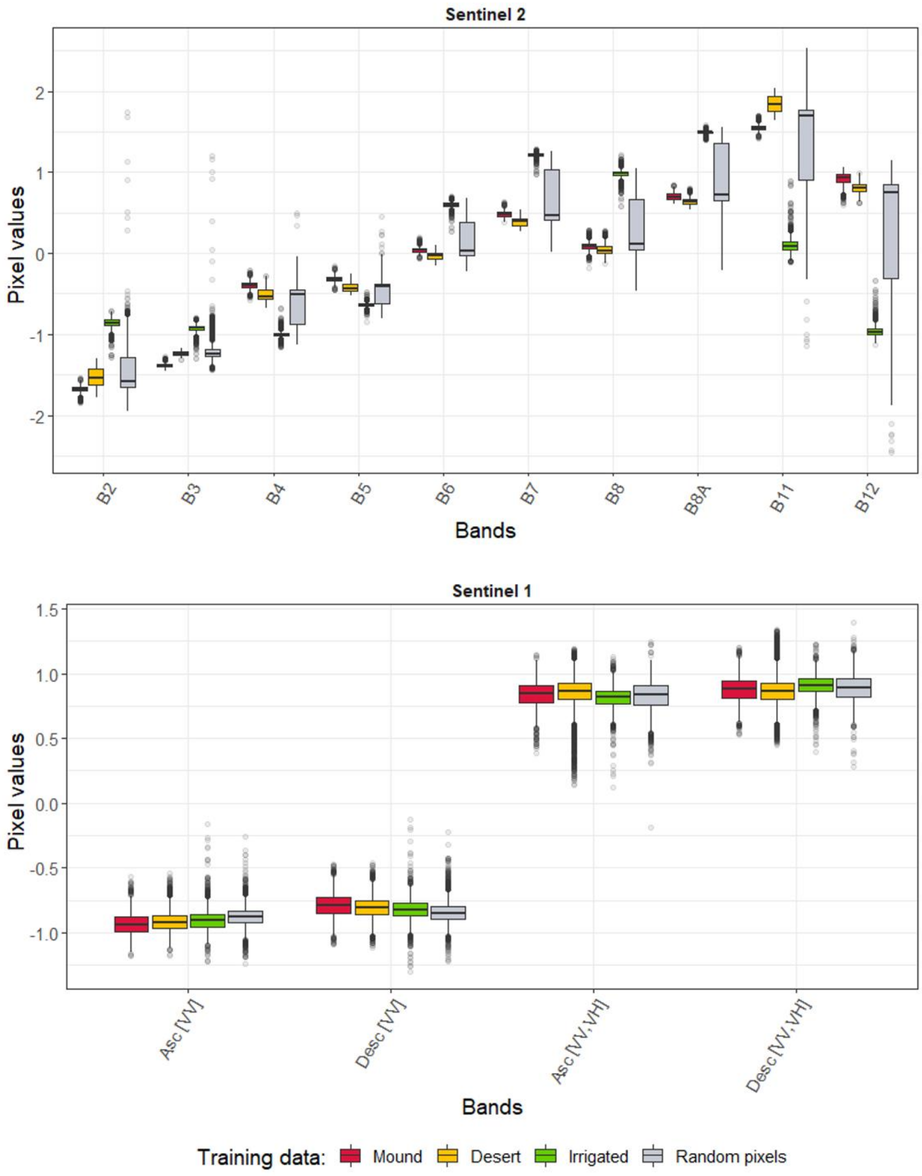

Figure S1. Multispectral (Sentinel 2) and SAR (Sentinel 1) signatures: values for “mound” pixels used as training set are compared to an additional dataset of land cover types in the region and a randomly distributed set of pixels.

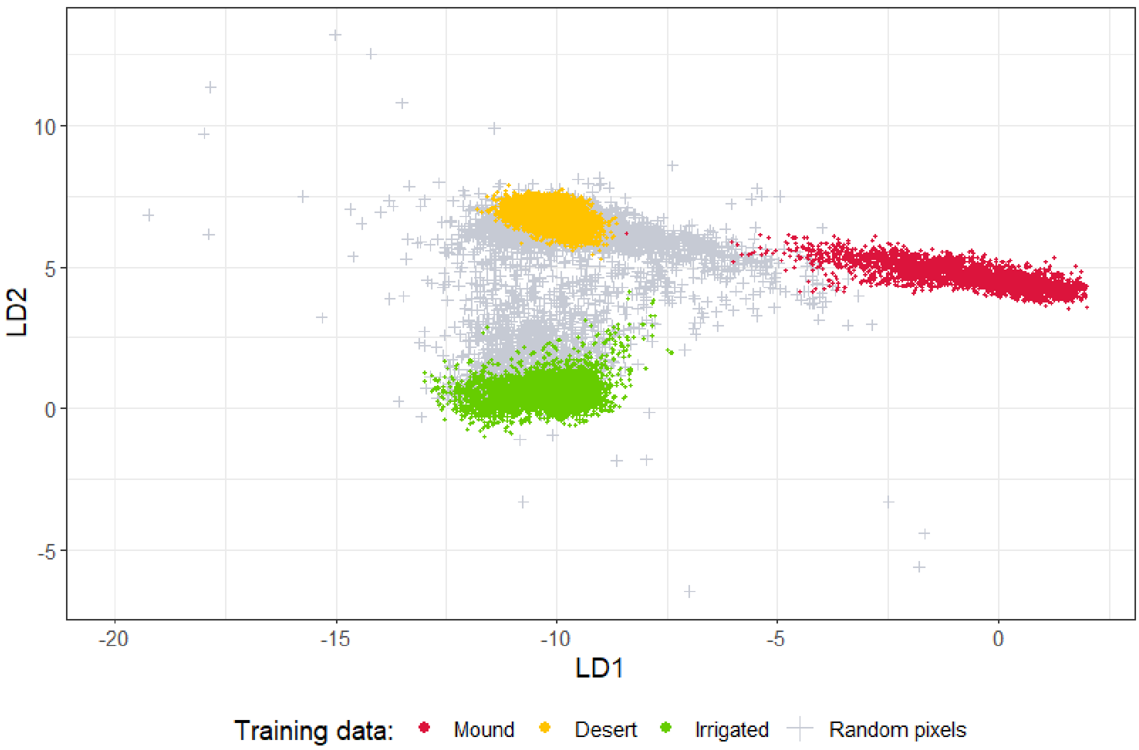

Figure S2. Linear Discriminant Analysis. Biplot indicating clear within-class vs. among-class covariance among the predefined training classes. The training set of "mound" pixels is clustered towards the right of the LDA ordination space, thus suggesting a robust distinctiveness of mound-like surfaces.

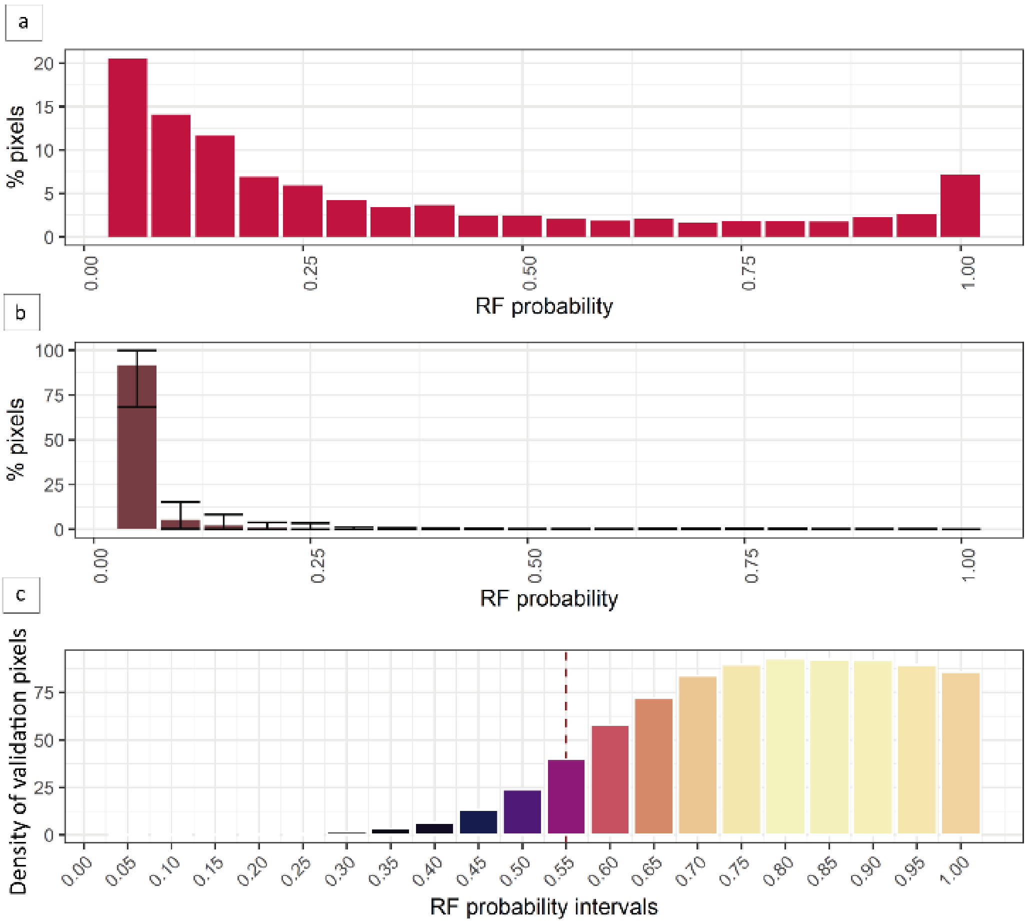

Figure S3. Validation set and RF probability for **a)** polygons used as “mound” validation set at their observed positions; and **b)** the same polygons at randomized positions (Monte Carlo simulation). In **c),** the density of validation “mound” pixels is represented by intervals of RF probability.

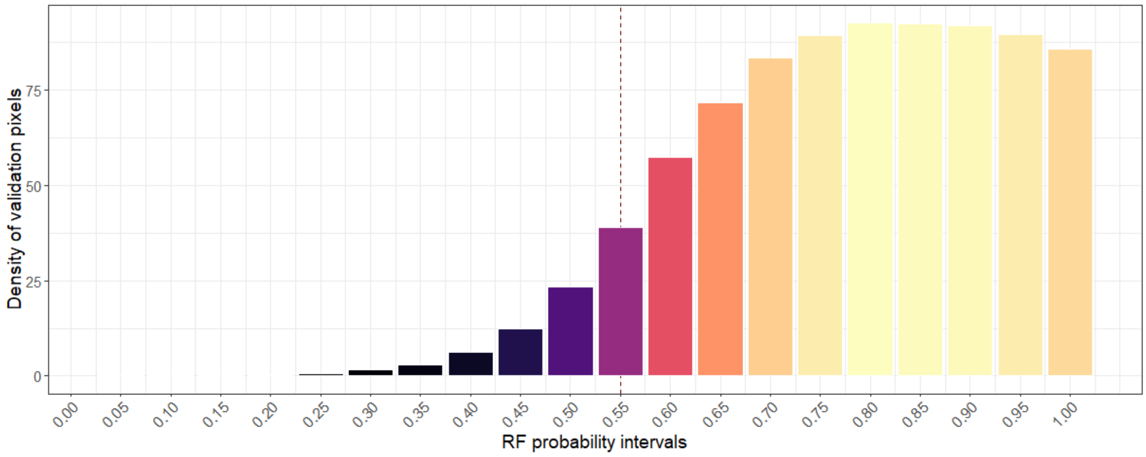

Figure S4. RF probability densities for all the digitalised pixels that are included in the final dataset of 337 mound-like areas. Note the robust higher density of archaeological mounds beyond the >0.55 RF probability threshold. Low RF probability values mostly represent photo-interpreted mounds highly affected by present day land cover.

References

1. R Core Team, *R: A Language and Environment for Statistical Computing* (R Foundation for Statistical Computing, Vienna, 2018). Available at: https://www.r-project.org.
2. W. N. Venables, B. D. Ripley, *Modern Applied Statistics with S* (Springer, New York, 2002).
3. G. James, D. Witten, T. Hastie, R. Tibshirani, *An Introduction to Statistical Learning - with Applications in R* (Springer, New York, 2013).
4. W. P. Megarry, G. Cooney, D. C. Comer, C. E. Priebe, Posterior probability modelling and image classification for archaeological site prospection: Building a survey efficacy model for identifying Neolithic felsite workshops in the Shetland Islands. *Remote Sens*. **8(6)**, 1–18 (2016).
5. F. C. Conesa *et al*., Multi-proxy survey of surface archaeological scatters in drylands: the case of North Gujarat, India. *Quat. Int*. **435**, 57–75 (2017).

Multitemporal multisource machine learning algorithm employed to obtain the result presented in the text.

```
// JavaScript code to be implemented in Google Earth Engine(c) developed by H.A. Orengo to
// accompany the paper:

// Orengo, H.A.; Conesa, F.C.; Garcia-Molsosa, A.; Lobo, A.; Green, A.S.; Madella, M. and Petrie,
// C.A. 2020. 'Automated detection of archaeological mounds using machine learning classification
// of multi-sensor and multi-temporal satellite data' submitted to PNAS.

//          ------------- ooo -------------

// TO EXECUTE THE ALGORITHM PASTE THIS CODE INTO GOOGLE EARTH ENGINE CODE EDITOR AND PRESS 'Run'

//          ------------- ooo -------------

// For more information on how this code works and how to apply it refer to the text of the article.
// Suggestions and code improvements are welcome! Please, contact Hector A. Orengo at horengo@icac.cat

// NOTES, READ CAREFULLY!
// 1. Visualisation parameters for each layer can be adjusted in the Layers dialogue in the map
//    area of Google Earth Engine.
// 2. To apply these analyses to your own areas:
//      1.  Define a central point in your study area (as X,Y WGS84 decimal degrees) and a scale
//          for visualisation purposes in line 51.
//      2.  Eliminate the polygon variable in lines 61-108 and draw your own polygon (as a geometry)
//          delimiting your own Area of Interest (AOI) using the Geometry Imports menu in the top
//          right corner of the map area below.
//      3.  Delete the 'sites' and 'other' variables in the 'Machine learning' section and create
//          two new polygon feature collections with the same names using the polygon drawing tool
//          in the top left map area. Remember to: a) make sure the new polygon layers are feature
//          collections; b) add property called 'class' with a value of one for the 'sites' layer
//          and a value of 0 for the 'other' layer; c) draw polygons delimiting sites you will use
//          as training data for the 'sites' layer and any other type of landcover present (such as
//          vegetation, desert, water, and so on) for the 'other' layer. After obtaining the results
//          of the classification use these results to select more training polygons to improve the
//          results in the next iterations of the algorithm.
// 3. The images resulting from this script can be transferred to your Assets or Google Drive running
//    the analysis from the 'Tasks' tab on the top right of the screen and selecting the Drive option.
// 4. No outputs of kernel-based results (filtering of single high-probability pixels and vectorisation
//    of the results) have been included in the map layer as EE executes these using the screen
//    resolution. To obtain the raster resulting from the morphologic filter and its vectorisation run
//    the analysis on the 'Tasks' tab. This sends the analysis to run on the server at full resolution.
//    Before running the filtering or vectorisation process read the -- WARNING -- in lines 249-255.
// 5. Specific instructions of how the algorithm works have been included within the code, please,
//    read these carefully and consult the paper's text to understand how to best apply it.


//          ------------- ooo -------------


// Define a central point in your study area (as X,Y WGS84 decimal degrees) and a scale,
// just for visualisation purposes. Change the coordinates to center the map in your own area
Map.setCenter(72.0428, 28.8447, 9);

// Indicate here iteration number and comments for naming the results
var iteration = 'it03';

// Create a polygon delimiting the AOI. In our case it is limited by the India-Pakistan border on
// its east side (as currently defined by EE).
// The user can just eliminate this polygon (the whole variable below) and draw his/her own polygon
// (as a geometry) delimiting a new AOI using the Geometry Imports menu in the top right
// corner of the map area below.
var geometry = /* color: #ffffff */ee.Geometry.Polygon(
        [[[72.45830862199341, 29.750552355244295],
          [71.56292287980591, 29.420943340410837],
          [71.01360647355591, 29.167036496936127],
          [70.50823537980591, 28.710347184314795],
          [69.81335012589966, 28.145148616022762],
          [69.81403677140747, 27.806470493744765],
          [70.13524030890267, 27.80691077970534],
          [70.14361534618399, 27.81924011961056],
          [70.19595210629575, 27.865568170389185],
          [70.22910634738344, 27.90336008925868],
          [70.30078110977706, 27.93859381059637],
          [70.36959504400818, 28.010891479325696],
          [70.43631096947502, 28.020339734987342],
```

```
          [70.50166169909733, 28.038239115709796],
          [70.55217408774433, 28.026853985776395],
          [70.58619708587139, 28.013031425772674],
          [70.59686024591633, 27.994368049466157],
          [70.67757741784033, 27.92112382776193],
          [70.67482388569022, 27.875304760850973],
          [70.68504668856156, 27.831124438046768],
          [70.73134544986533, 27.760441143885107],
          [70.73799881516959, 27.744538198666564],
          [70.7611263284465, 27.721341098010463],
          [70.78838123782589, 27.716671141396315],
          [70.87299856497816, 27.704965787566607],
          [70.90542874721541, 27.711473892612446],
          [70.96206249621889, 27.73053659203472],
          [71.00032589621992, 27.748971687642825],
          [71.20075266566528, 27.83535362519099],
          [71.38139621599032, 27.872048361937328],
          [71.66534371633725, 27.879391737019766],
          [71.89597316155891, 27.96174525590272],
          [71.93064543920536, 28.12628319926123],
          [72.00685697697509, 28.222437609592077],
          [72.13421194230591, 28.316959172219075],
          [72.20704561235095, 28.39904278337407],
          [72.30060300945013, 28.67054467287233],
          [72.35306763729557, 28.73223060323902],
          [72.40490211802103, 28.78305813071367],
          [72.48232155059634, 28.813692704333132],
          [72.7344705855187, 28.94884380122061],
          [72.94630488645794, 29.02877070048368],
          [73.00350515519654, 29.153844875278295],
          [73.08520807734783, 29.235790953385802],
          [73.10989574272867, 29.283124550218936],
          [72.98565237199341, 29.42572796212171],
          [72.76592580949341, 29.717162871938747]]]);

print('Study area', geometry.area().divide(1000 * 1000), 'km2'); // AOI area km2 for info


// ////////////////////// IMPORT & COMPOSITE SENTINEL 1 COLLECTION ////////////////////////

// Load the Sentinel-1 ImageCollection
var s1 = ee.ImageCollection('COPERNICUS/S1_GRD')
  .filterBounds(geometry)
  .filterDate('2014-10-03', '2020-06-05');

// Print total Sentinel 1 images employed
print('Sentinel 1 images:', s1);

// Filter to get images from different look angles
var asc = s1.filter(ee.Filter.eq('orbitProperties_pass', 'ASCENDING'));
var desc = s1.filter(ee.Filter.eq('orbitProperties_pass', 'DESCENDING'));
var vvvhAsc = asc
  // Filter to get images with VV and VH single polarization
  .filter(ee.Filter.listContains('transmitterReceiverPolarisation', 'VV'))
  .filter(ee.Filter.listContains('transmitterReceiverPolarisation', 'VH'))
  // Filter to get images collected in interferometric wide swath mode.
  .filter(ee.Filter.eq('instrumentMode', 'IW'));
var vvvhDesc = desc
  // Filter to get images with VV and VH dual polarization
  .filter(ee.Filter.listContains('transmitterReceiverPolarisation', 'VV'))
  .filter(ee.Filter.listContains('transmitterReceiverPolarisation', 'VH'))
  // Filter to get images collected in interferometric wide swath mode.
  .filter(ee.Filter.eq('instrumentMode', 'IW'));

// Create a composite from means at different polarizations and look angles.
var composite = ee.Image.cat([
  vvvhAsc.select('VV').median(),
  vvvhAsc.select('VH').median(),
  vvvhDesc.select('VV').median(),
  vvvhDesc.select('VH').median(),
]).clip(geometry);

// Rename the bands so you can identify them when they are all joined together
var s1comp = composite.select(
    ['VV','VH','VV_1','VH_1'], // old names
    ['s1vva','s1vha','s1vvd','s1vhd'] // new names
);


// ////////////////////// IMPORT & COMPOSITE SENTINEL 2 COLLECTION ////////////////////////

// Function to mask clouds using the Sentinel-2 QA band.
function maskS2clouds(image) {
  var qa = image.select('QA60');
```

```
  // Bits 10 and 11 are clouds and cirrus, respectively.
  var cloudBitMask = 1 << 10;
  var cirrusBitMask = 1 << 11;

  // Both flags should be set to zero, indicating clear conditions.
  var mask = qa.bitwiseAnd(cloudBitMask).eq(0).and(
             qa.bitwiseAnd(cirrusBitMask).eq(0));

  // Return the masked and scaled data, without the QA bands.
  return image.updateMask(mask).divide(10000)
      .select("B.*")
      .copyProperties(image, ["system:time_start"]);
}

// Map the function over one year of data and take the median.
// Load Sentinel-2 TOA reflectance data.
var S2_col = ee.ImageCollection('COPERNICUS/S2')
    .filterBounds(geometry)
    .filterDate('2015-06-23', '2020-06-05')
    // Pre-filter to get less cloudy granules.
    .filter(ee.Filter.lt('CLOUDY_PIXEL_PERCENTAGE', 20))
    .map(maskS2clouds);

// Print total Sentinel 2 images employed
print('Sentinel 2 images', S2_col);

// Select the bands of interest form the Image Collection
var s2comp = S2_col.select(['B2','B3','B4','B5','B6','B7','B8','B8A','B11','B12'])
  .mean().clip(geometry);


// ///////////////////// COMPOSITE SENTINEL 1 & 2 MEAN BANDS ///////////////////////

// Join the S1 and S2 composites in a single composite that will be used for the extraction
// of training data and the application of the trained classifier
var fullComposite = ee.Image([s1comp, s2comp]);

// Reduction in the number of decimal places of the values of the resulting raster
// This will not reduce noticeably the quality of the data but it will reduce significantly
// the size of the resulting raster.
var Composite = ee.Image(0).expression(
    'round(img * 10000) / 10000', {
      'img': fullComposite
    });

print('Composite:', Composite);


// ///////////////////// MACHINE LEARNING RF CLASSIFIER ///////////////////////

// Call training data for current iteration (in this case iteration 3). The user wanting to
// generate her/his own training data is prompted to use the geometry imports panel in the map
// view below to create new feature collections (named 'sites' and 'other' if the user wants to
// reuse the code below) with a property named 'class' and a value of 1 and 0 respectively.
// These will be used to identify known sites and all other types of landcover that do not
// correspond to sites.
var sites = ee.FeatureCollection('users/hao23/cholistan/sites_it3'),
    other = ee.FeatureCollection('users/hao23/cholistan/other_it3');

// Merge training data
var trn_pols = sites.merge(other);
print(trn_pols, 'train_pols');

// Create variable for bands
var bands = ['s1vva','s1vha','s1vvd','s1vhd','B2','B3','B4','B5','B6','B7','B8','B8A','B11','B12'];

// SampleRegions to extract band values for each pixel in each training polygon
var training = Composite.select(bands).sampleRegions({
  collection: trn_pols,
  properties: ['class'],
  scale: 10
});

// Apply RF classifier calling mode "probability"
var classifier = ee.Classifier.randomForest({'numberOfTrees':128})
  .setOutputMode('PROBABILITY').train({
  features: training,
  classProperty: 'class',
  inputProperties: bands
});

// Create classified probability raster
var classified = Composite.select(bands).classify(classifier);

// Add the resulting classified layer to the Map Window below
```

```
Map.addLayer(classified, {min: 0.55, max: 1}, iteration); // It can take several minutes to load


// ////////////////////// FILTER OF SMALL FALSE POSITIVES ////////////////////////
// WARNING: This part of the code is included but not used in the paper. Many mounds are partially buried by sand
// and present elongated shapes that could be filtered out as they do not present enough contiguous high-probability
// pixels. Only use the filtering process in areas where your features (in this case mounds) are larger than the
// selected kernel size.
// ALSO: the vectorisation process can produce an error related to the projection of the data or lack of memory
// this can be solved by exporting the filtered layer as an asset and apply the filter to the asset directly without
// running all the code above.

// Threshold of the percentage of the RF classification
var gt = 0.55; // Probability value selected as threshold
var threshold = classified.select('classification').gt(gt);

// Median filter to eliminate isolated high probability pixels
var r = 1; // Radius (in pixels) selected for the kernel
var filtered = threshold.focal_median({
		kernel: ee.Kernel.square({radius: r, units: 'pixels'})
}).gt(0);


// ////////////////////// VECTORISATION OF THE FILTERED RASTER ////////////////////////

var vectors = filtered.mask(filtered).reduceToVectors({geometryType: 'polygon', maxPixels: 9e12});


// ////////////////////// EXPORT OF RESULTING DATASETS ////////////////////////

// Data exports as assets so they can be included and visualises in next iterations
Export.image.toAsset({ // It is also possible to export to Google Drive, just select the option in the dialogue
  image: classified,
  description: 'rf128_S1-S2_prob_' + iteration,
  scale: 10,
  maxPixels: 1e12,
  region: geometry
});

// Before using make sure you have read the WARNING in 249-255
Export.image.toAsset({ // Also possible to export to Google Drive, select when dialogue appears
  image: filtered,
  description: 'rf128_s1-s2_prob_' + iteration + '_filter' + Math.round(gt*100) + '_med_r' + r,
  scale: 10,
  maxPixels: 1e12,
  region: geometry
});

// This vector layer employs the results of the filtering process, before exporting and using the results make sure
// you have read the WARNING in lines 249-255
Export.table.toAsset({ // Also possible to export to Google Drive, select when dialogue appears
  collection: vectors,
  description:'vector_rf128_s1-s2_prob_' + iteration + '_filter' + Math.round(gt*100) + '_med_r' + r,
});
```
